# Identification of chronological and photoageing-associated microRNAs in human skin

**DOI:** 10.1038/s41598-018-31217-8

**Published:** 2018-08-28

**Authors:** Ankit Srivastava, Magnus Karlsson, Claire Marionnet, Françoise Bernerd, Audrey Gueniche, Charles E. l. Rawadi, Mona Ståhle, Enikö Sonkoly, Lionel Breton, Andor Pivarcsi

**Affiliations:** 1 Department of Medicine Solna, Dermatology and Venereology section, Karolinska Institutet, Stockholm, Sweden; 2 L’Oréal Research and Innovation, Aulnay-sous-Bois, France; 3 Unit of Dermatology, Karolinska University Hospital, Stockholm, Sweden

## Abstract

MicroRNAs are short non-coding RNAs that play key roles in regulating biological processes. In this study, we explored effects of chronological and photoageing on the miRNome of human skin. To this end, biopsies were collected from sun-exposed (outer arm, n = 45) and sun-protected (inner arm, n = 45) skin from fair-skinned (phototype II/III) healthy female volunteers of three age groups: young, 18–25 years, middle age, 40–50 years and aged, > 70 years. Strict inclusion criteria were used for photoageing scoring and for chronological ageing. Microarray analysis revealed that chronological ageing had minor effect on the human skin miRNome. In contrast, photoageing had a robust impact on miRNAs, and a set of miRNAs differentially expressed between sun-protected and sun-exposed skin of the young and aged groups was identified. Upregulation of miR-383, miR-145 and miR-34a and downregulation of miR-6879, miR-3648 and miR-663b were confirmed using qRT-PCR in sun-exposed skin compared with sun-protected skin. qRT-PCR analysis revealed that miR-383, miR-34a and miR-134 were differentially expressed in all three age groups both in chronological and photoageing, suggesting a synergetic effect of intrinsic and extrinsic ageing on their expression. In conclusion, our study identifies a unique miRNA signature which may contribute to skin ageing.

## Introduction

Skin ageing is a complex process leading to decrement of cutaneous structures and functions with time. Decline in regenerative potential, impaired epidermal barrier function, decrease in resistance to infections and impairment of mechanical properties such as loss of elasticity and extensibility are key markers of skin ageing^1,2^. Skin ageing involves two different processes: intrinsic (chronological) ageing, primarily determined by genetic factors, and extrinsic (photoageing) primarily due to solar UV exposure. The chronological ageing of human skin is characterized by increased laxity, fine wrinkling and dermal atrophy with reduced amounts of type I and III fibrillar collagens^1,2^. The extrinsic ageing of the skin is mainly caused by chronic solar exposure, which leads to alterations in biomolecules such as DNA, RNA and proteins. Phenotypicaly, the skin undergoes wrinkling, dischromia, assumes a leathery appearance and develops telangiectasia. The main feature of photoageing is the formation of solar elastosis, an accumulation of abnormal elastotic material together with an amplified reduction in type I and III collagens, and the presence of dermal infiltrates^1,3^. Furthermore, cumulative exposure to solar UV radiation can lead to the development of non-melanoma skin cancer.

MicroRNAs (miRNAs) are a group of small noncoding RNAs (~22 nucleotides), that negatively regulate gene expression at the post-transcriptional level via binding to the 3′-untranslated region (3′-UTR) of their target mRNAs^4,5^. In human skin, miRNAs are involved in epidermal development, proliferation, differentiation^6–11^, inflammatory responses, immune regulation^12^, as well as in wound healing^13,14^. Altered miRNA expression is also associated with skin diseases such as psoriasis and atopic dermatitis, and the development of melanoma and non-melanoma skin cancers.

miRNAs have recently emerged as important regulators of cellular senescence and ageing in various organisms. In *C*. *elegans*, miRNAs have been shown to regulate the lifespan of the adult animals, demonstrating their important role in ageing^15^. Recent studies comparing expression levels of miRNAs in young and old tissues in mice and humans have identified a number of miRNAs which are differentially expressed during ageing in mammals^15–18^. Several studies investigated the involvement of miRNAs in human skin ageing although most of them were performed in *in vitro* systems such as senescent fibroblasts and keratinocytes or artificially aged cells using UV treatment^19,20^ and their *in vivo* relevance is not yet known. The aim of the present study was to explore the impact of chronological ageing and photoageing on the miRNome of human skin.

## Results

### miRNA profiling identifies robust alterations in miRNA expression in photoaged skin

To investigate changes in the miRNome of the skin during photoageing and chronological ageing, RNA samples were obtained from the outer and the inner arm, representing sun-exposed and sun-protected skin, respectively, of healthy young (age group #1, 18–25 years), middle age (age group #2, 40–50 years) and aged donors (age group #3, > 70 years). In order to decrease the possible variation due to differences in skin pigmentation among the donors, volunteers with similar skin type (individual typology angle, ITA: average ITA of 37.9 for groups #1 and #2 and 37.8 for age group #3, phototype II to III) were selected for inclusion to the study (Table 1 and Supplementary Table S1). To match the biological and chronological age of donors, skin folding grade was assessed with Densiscore®. We observed numerous thin folds in young donors and fewer wider folds in old donors corresponding to an increased skin folding grade with increasing age (average 1 for age group #1, 3 for age group #2 and > 6 for age group #3) (Table 1 and Supplementary Table S1). Moreover, the photoageing score was measured on the sun-exposed outer arm of the volunteers using a photographic scale. The average photoageing score was 1.2 for age group #1, 4.2 for age group #2 and 6.2 for age group #3 (Table 1 and Supplementary Table S1). For detailed inclusion and exclusion criteria, see supplementary methods.

**Table 1:** Summary of donor characteristics in the three age groups.

Age group	Mean age	Mean ITA value	Mean skin fold	Mean photoaging grade
Age group #1 (18–25 years)	22 ± 1.91	37.9 ± 4.11	1,0	1.2 ± 0.41
Age group #2 (40–50 years)	44.7 ± 2.67	37.9 ± 3.38	3,0	4.2 ± 0.41
Age group #3 (>70 years)	74.3 ± 3.61	40.3 ± 2.54	>6	6.2 ± 0.41

To identify differentially expressed miRNAs during the chronological ageing of the skin, we compared the miRNA expression profile of sun-protected skin samples from age group #1 and age group #3 by microarray-profilling, which could detect changes in the expression of 2578 miRNAs. No statistically significant (False Discovery Rate, (FDR) < 5%) effect of age on miRNA expression was detected, suggesting that chronological ageing in itself has little effect.

**Figure 1:**
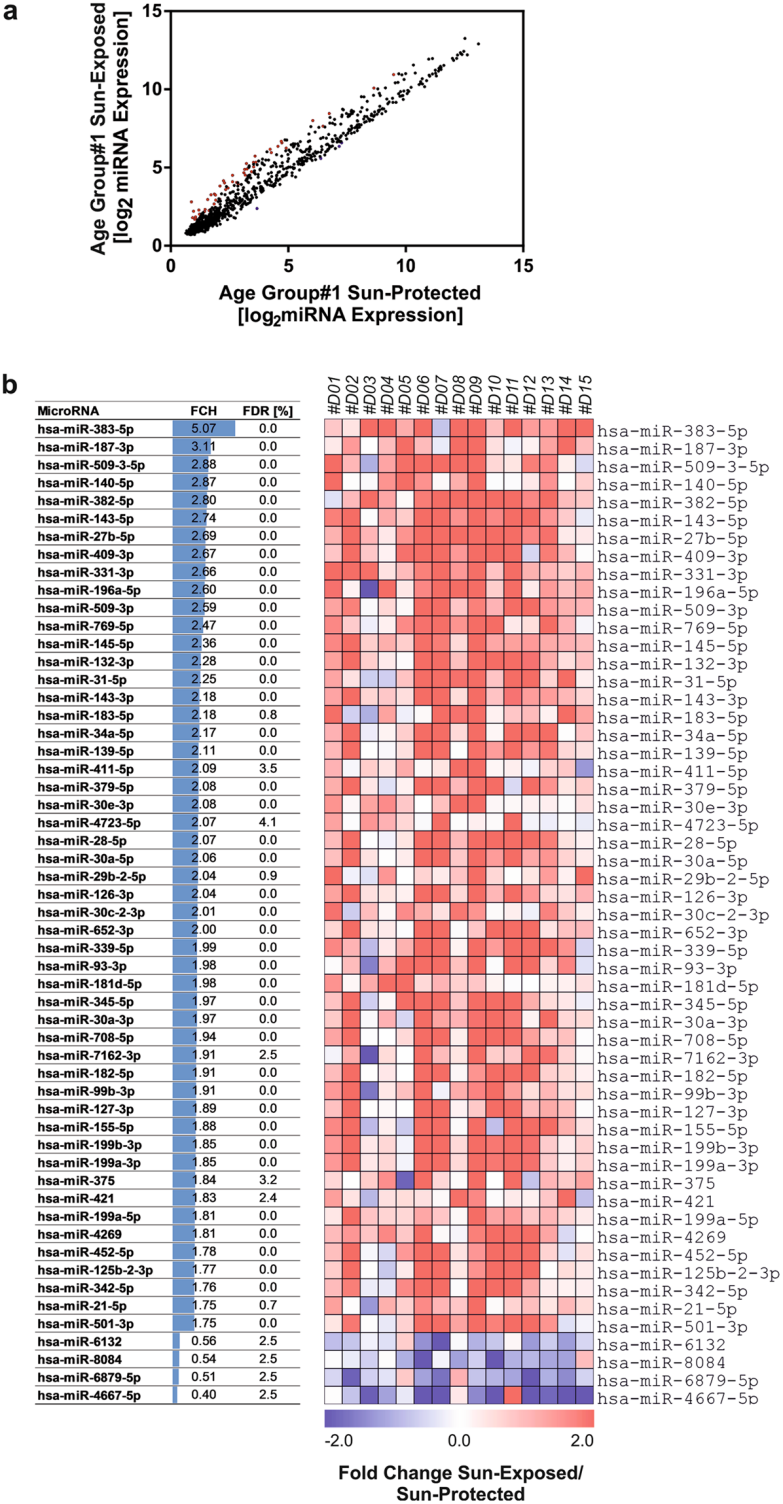
Identification of differentially expressed miRNAs between sun-protected and sun-exposed skin of young individuals (age group 1). Total RNA was isolated from the skin biopsies taken from the outer and the inner arm of young donors (age group #1, 18–25 years) representing sun-exposed and sun-protected skin, respectively. miRNA expression was analyzed by microarray containing 2578 miRNA probes. 55 miRNAs were differentially expressed between inner and outer arm skin in age group #1 (FDR < 5%). (**a**,**b**) Heatmap and the table displaying the fold change of differentially expressed miRNAs in sun-exposed skin compared to sun-protected skin.

To determine whether there are any differences in the miRNAs expression profile of sun-protected and sun-exposed skin, we next performed pair-wise analysis of miRNA expression in skin samples of age group #1, collected from the inner and outer arm of donors. A robust difference in miRNA-profiles was observed (Fig. 1a,b) suggesting early molecular changes due to sun exposure, which may prelude photoageing. Fifty-five miRNAs were significantly altered in sun-exposed skin (FDR < 5%), 94% (51/54) of which were up-regulated in the sun-exposed condition.

**Figure 2:**
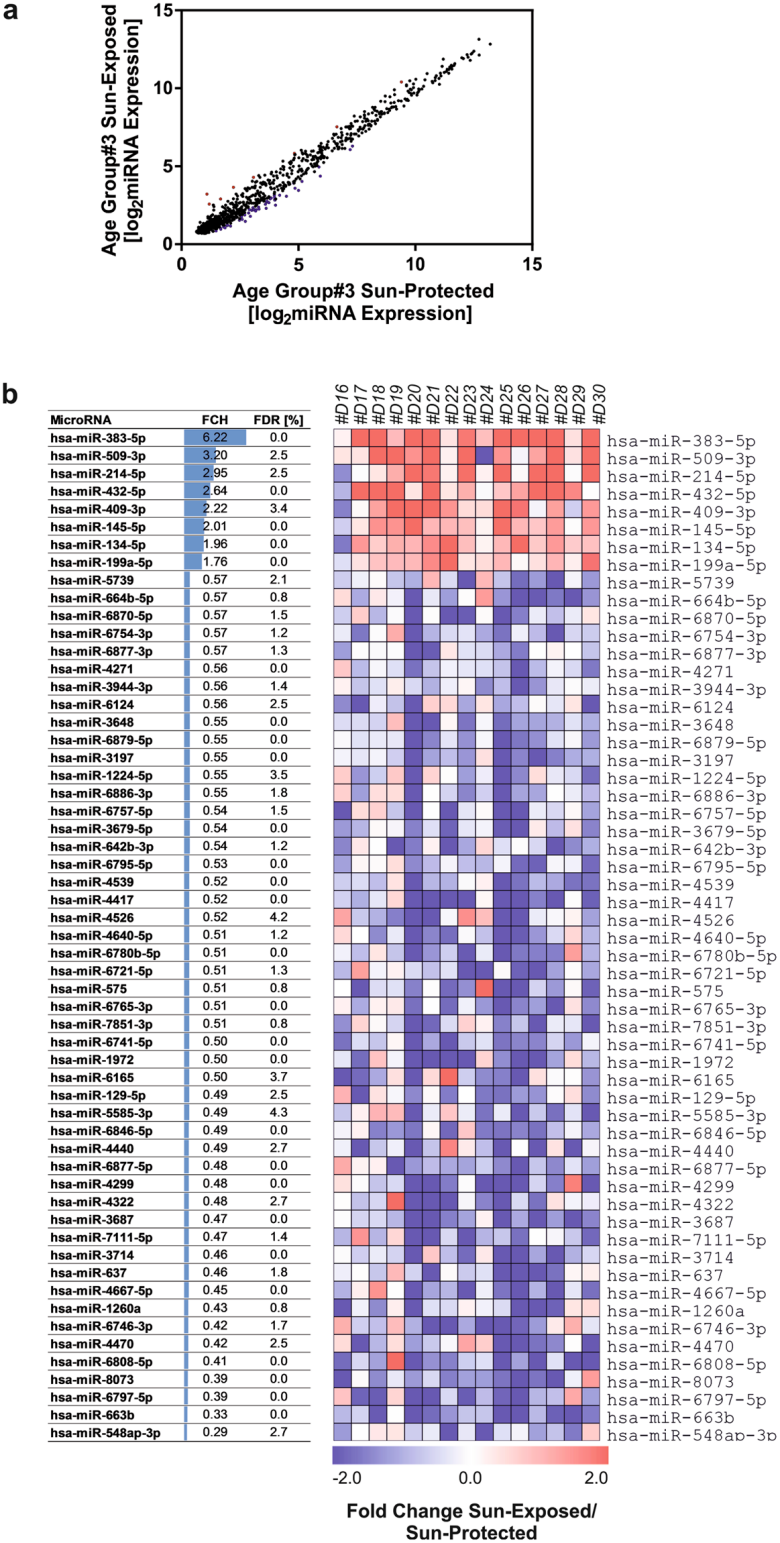
Identification of differentially expressed miRNAs between sun-protected and sun-exposed skin in aged individuals (age group #3). Total RNA was isolated from the skin biopsies taken from the sun-exposed and sun-protected skin of aged donors (age group #3, > 70 years). miRNA expression profiling identified 57 differentially expressed miRNAs between inner and outer arm skin of donors in age group #3 (FDR < 5%). (**a**,**b**) Heatmap and the table displaying the fold change of differentially expressed miRNAs in sun-exposed skin compared to not sun-exposed skin.

We next extended the analysis and compared miRNA expression between sun-exposed and sun-protected skin samples obtained from donors in age group #3 (>70 years). Fifty seven miRNAs were differentially expressed between sun-protected and sun-exposed skin (FDR < 5%). In contrast to the results in age group #1, most of the deregulated miRNAs in age group #3 were downregulated (49/57; 86%) and only 8 (14%) were upregulated (Fig. 2a,b). These results suggest that sun exposure may have a higher impact at the level of miRNAs expression compared to chronological ageing in young as well as in old age skin.

**Figure 3:**
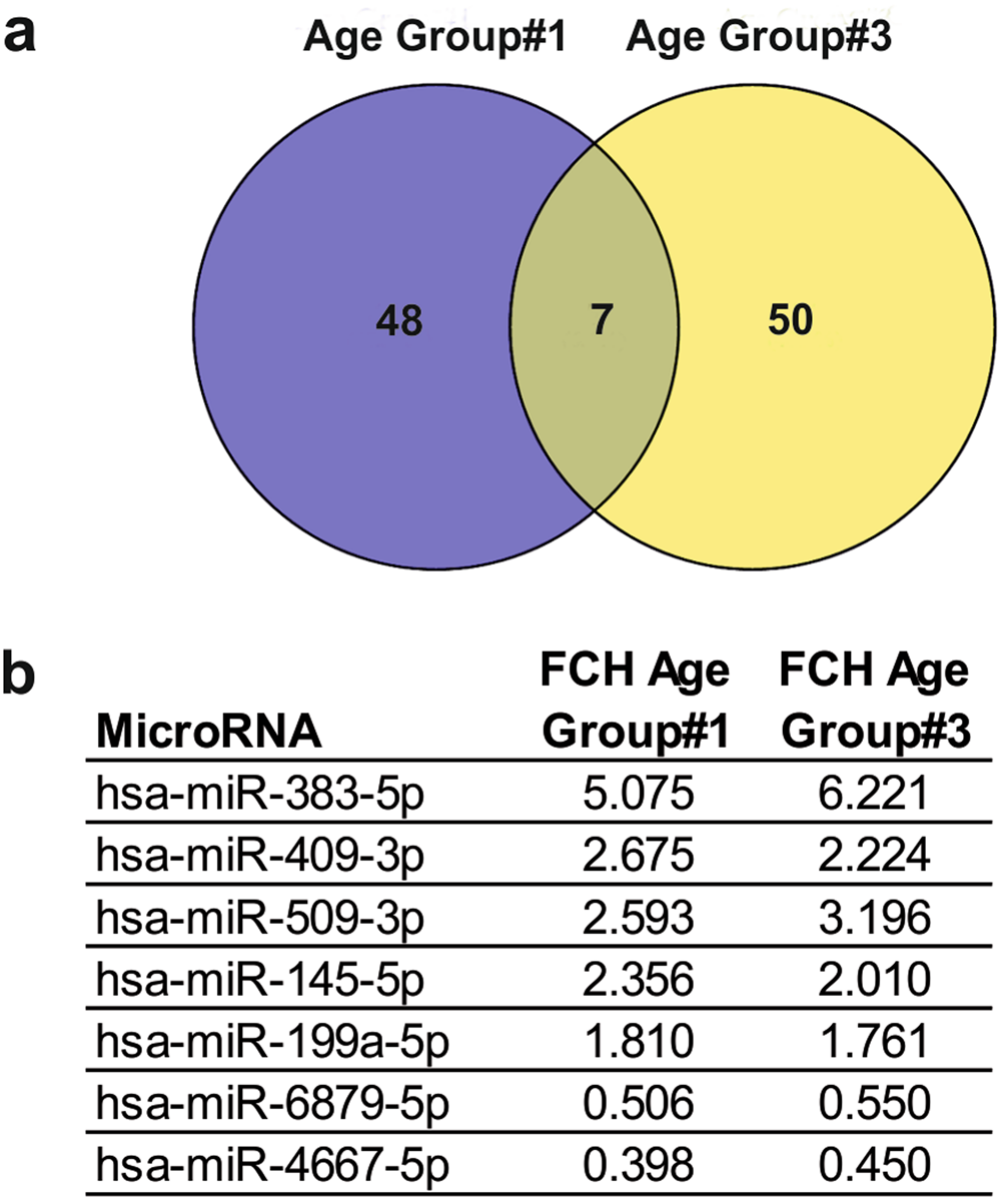
Venn-diagram identifying miRNAs that were altered in age groups #1 and #3 between sun-protected and sun-exposed skin. An intersection was performed among the differentially expressed miRNAs in sun-exposed skin of young and old age group using an online tool Venny 2.1. (**a**,**b**) Seven miRNAs were found common among the differentially expressed miRNAs in young and aged individuals. The changes in the expression of these miRNAs were in the same direction in both age groups (5 upregulated and 2 downregulated).

In an attempt to identify miRNAs whose level is consistently associated with sun exposure, we compared the lists of photo-exposure-regulated miRNAs in the young and old age groups (Fig. 3a,b). The intersection analysis of miRNAs, whose expression was significantly altered in both groups identified 7 miRNAs, out of which 5 were upregulated (miR-383, miR-409, miR-509, miR-145 and miR-199a) and 2 were downregulated (miR-6879 and miR-4667) in sun-exposed skin samples (Fig. 3a,b). These 7 miRNAs constitute a core-set of miRNAs which may reflect the overall impact of photo-exposure on the skin irrespective of age.

To identify the combined impact of solar exposure and chronological ageing on the miRNA signature of young and old donors, we compared the miRNA expression profile of sun-exposed skin of age group #1 to that of age group #3. 17 miRNAs were differentially expressed in sun-exposed skin of age group #3 (FDR < 20%) compared to age group #1. Fourteen of them were upregulated and 3 of them were downregulated (Supplementary Figure S1a,b).

### Validation of differentially expressed miRNAs in photo and chronologically aged donors with qRT-PCR

**Figure 4:**
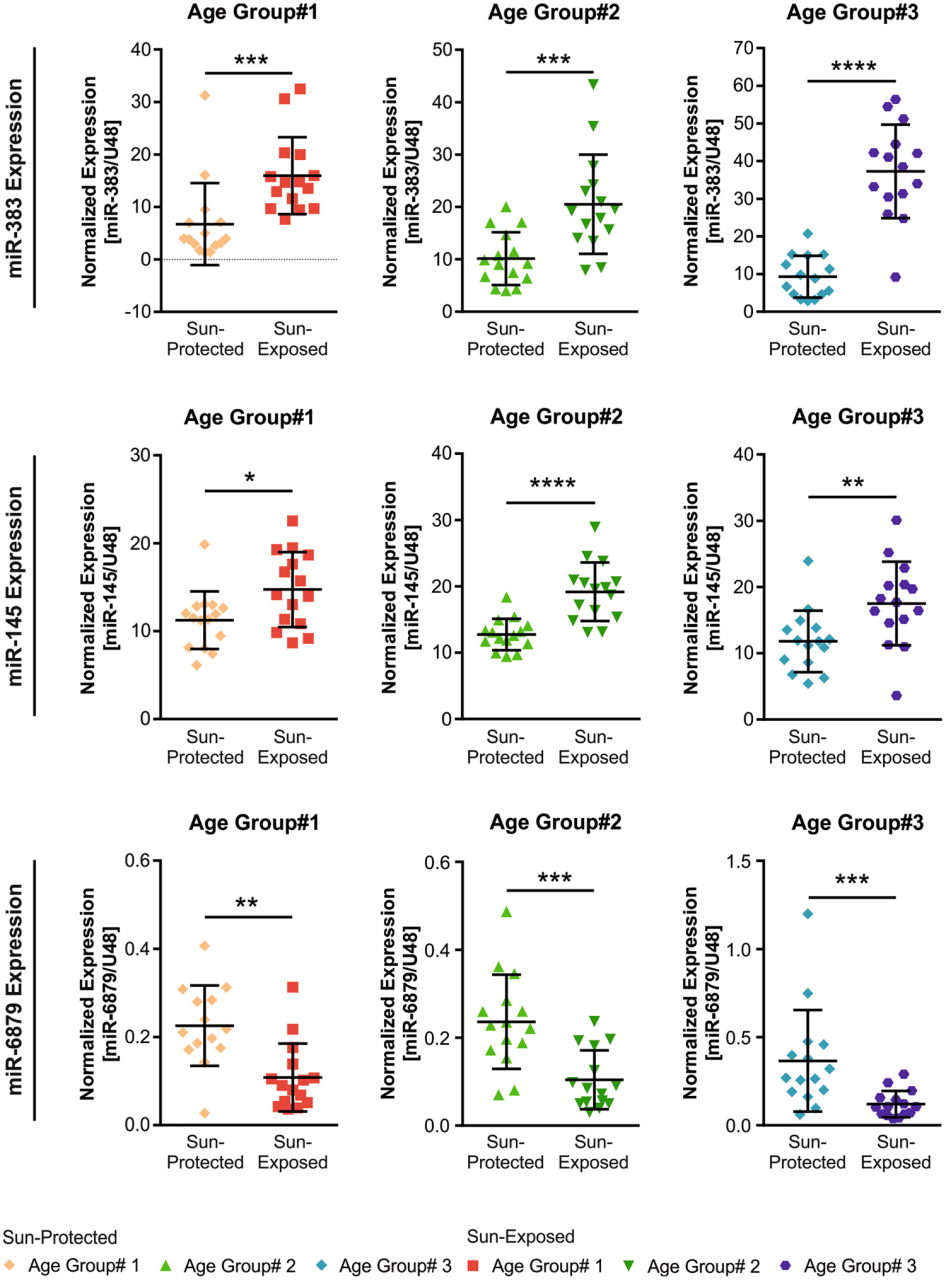
qRT-PCR validation of miRNAs whose expression was altered in both age group #1 and #3. The expressions of miR-383, miR-145 and miR-6879 were analyzed using qRT-PCR in skin samples collected from sun-exposed (n = 45) or sun-protected (n = 45) zones of all the 3 age groups. miR-383 and miR-145 were upregulated while miR-6879 was downregulated in skin samples collected from the outer arm (sun-exposed) in all three age groups compared with paired skin samples that were collected from the inner arm (sun-protected). Data are expressed in normalized units compared to U48 RNA. Error bars represent the standard deviation from the mean. ****p < 0.0001, ***p < 0.001, **p < 0.01, *p < 0.05. Mann-Whitney U test.

Next, we aimed to validate the microarray results by qRT-PCR using skin samples obtained from sun-exposed and sun-protected skin from young, middle age and old donors (Table 1; Fig. 4). Five miRNAs, which were deregulated in sun-exposed skin were selected for validation: miR-383, which was the top most upregulated miRNA in age groups #1 and #3 (Figs 1b, 2b), miR-145-5p, miR-409-3p and miR-199a-5p which were upregulated and miR-6879 which was downregulated. In accordance with the array data, qRT-PCR analysis of miR-383 and miR-145 confirmed their upregulation in the sun-exposed skin of age group #1 and #3 compared with sun-protected skin. Moreover, they were also upregulated in sun-exposed skin of age group #2 compared with sun-protected skin (Fig. 4). In line with the results of the microarray profiling, qRT-PCR analysis confirmed the decreased expression of miR-6879 in skin samples collected from sun-exposed area compared with paired skin samples that were collected from the sun-protected area in all three age groups. The qRT-PCR results obtained for miR-409-3p and miR-199a-5p did not confirm the microarray results (*data not shown*) highlighting the importance of validation experiments for microarray-studies.

**Figure 5:**
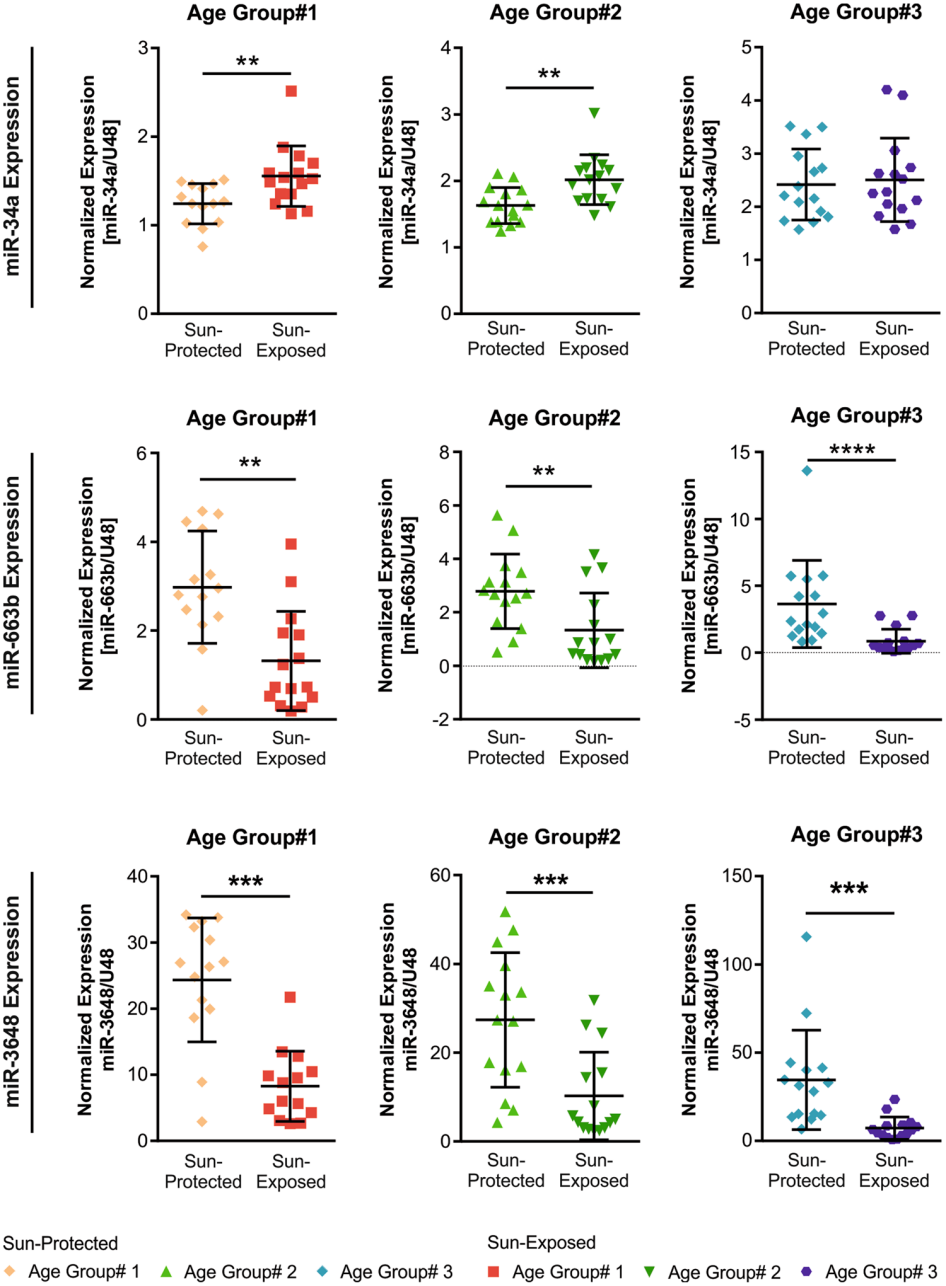
qRT-PCR-validation of miRNAs that were regulated specifically in age group #1 or #3. The expressions of miR-34a, miR-663b and miR-3648 were analyzed using qRT-PCR in skin samples collected from sun-exposed (n = 45) or sun-protected (n = 45) zones of all the 3 age groups. miR-34a was upregulated while miR-663b and miR-3648 were downregulated in skin samples collected from sun-exposed skin in all three age groups as compared with paired skin samples that were collected from sun-protected skin. Data are expressed in normalized units compared to U48 RNA. Error bars represent the standard deviation from the mean. ****p < 0.0001, ***p < 0.001, **p < 0.01. Mann-Whitney U test.

Our profiling study also identified miRNAs, which were differentially expressed specifically in age group #1 (48 miRNAs) or in age group #3 (50 miRNAs) between sun-protected and sun-exposed skin (Fig. 3a,b). Among these, we selected three miRNAs for qRT-PCR validation (miR-34a, miR-143 and miR-769), which were increased in sun-exposed skin compared with sun-protected skin of young donors and three miRNAs (miR-663b, miR432, miR-3648), which were identified by the screen in the old age donors. In accordance with the results of the microarray, qRT-PCR analysis of miR-34a expression demonstrated that its level was increased in skin samples collected from sun-exposed skin compared with sun-protected skin samples in age group #1 (Fig. 5). Moreover, increased level of miR-34a was observed even in age group #2 (Fig. 5). In line with the microarray results, no significant differences could be observed in miR-34a levels in skin samples collected from age group #3 (Fig. 5).

 The qRT-PCR analysis demonstrated a significant decrease in miR-663b and miR-3648 in sun-exposed skin samples compared with sun-protected skin in all age groups studied (Fig. 5). These results indicate that miR-663b and miR-3648 modulation is not restricted to the old age but can be observed at all ages, although the modulation seems to be aggravated by ageing. The differential expression of miR-143, miR-769 and miR-432 detected by microarray was not validated by qRT-PCR.

**Figure 6:**
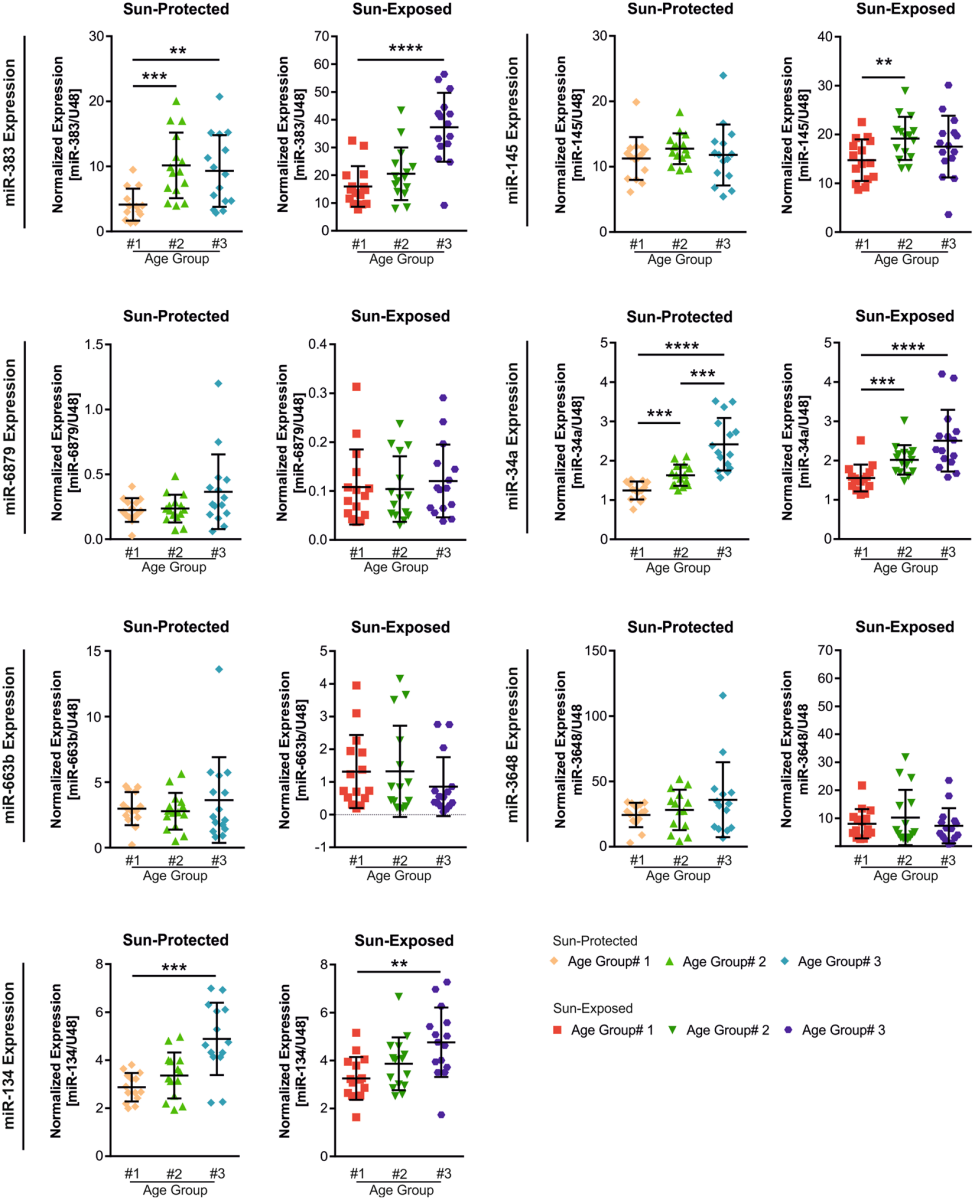
Analysis of the 7 ageing-associated miRNAs in the three age groups in sun-exposed and sun-protected skin sites. The expressions of miR-383, miR-145, miR-6879, miR-34a, miR-663b, miR-3648 and miR-134 were analyzed using qRT-PCR in the skin samples collected from sun-exposed (n = 45) or sun-protected (n = 45) zones and compared among the different age groups. Data are expressed in normalized units compared to U48 RNA. Error bars represent the standard deviation from the mean. ****p < 0.0001, ***p < 0.001, **p < 0.01. Mann-Whitney U test.

To identify the possible effect of chronological ageing on the validated miRNAs, we re-analyzed the qRT-PCR data according to the anatomical site, representing sun-protected and sun-exposed skin areas. qRT-PCR analyses of the seven selected miRNAs (miR-383, miR-145, miR-6879, miR-34a, miR-663b, miR-3648 and miR-134) revealed that the levels of miR-383 and miR-34a were significantly increased even in the sun-protected skin biopsies in age group #2 and #3 compared with age group #1 suggesting that the expression of these miRNAs were also altered during chronological ageing (Fig. 6). Moreover, the expression of miR-383 and miR-34a was increased in skin samples obtained from the sun-exposed skin of old age donors compared to sun-exposed skin of young donors (Fig. 6). In addition, miR-134 was also found up-regulated both during chronological and photoageing, as it was upregulated in age group #3 compared to age group #1, irrespective of solar exposure (Fig. 6). These findings indicate that the upregulation of miR-383, miR-34a and miR-134 in skin is associated with both chronological and photoageing and that the two may act in synergy. We were not able to observe any impact of chronological ageing on the expression of miR-145, miR-6879, miR-663b and miR-3648, confirming that these miRNAs are not influenced by chronological aging, and are only modulated by photo-exposure.

To explore which of the cells in skin express the validated miRNAs, we retrieved data from the atlas of miRNA expression across primary cells (FANTOM5 miRNA atlas: http://fantom.gsc.riken.jp/5/suppl/De_Rie_et_al_2017/vis_viewer/#/human#srna;miRNA)^21,22^ for miR-383, miR-145, miR-6879, miR-34a, miR-663b, miR-3648 and miR-134-expression in keratinocytes and fibroblasts. According to small RNA sequencing, miR-383 and miR-145 were expressed in fibroblasts but not detected in keratinocytes. miR-34a and miR-134 were expressed both in keratinocytes and fibroblasts (Supplementary Figure S2). The expression of miR-6879, miR-3648 and miR-663b was not detected in fibroblasts or keratinocytes in FANTOM5 miRNA atlas raising the possibility that these miRNAs are only expressed under specific *in vivo* conditions.

### Enrichement of biological pathways among the predicted targets of the validated miRNAs

To infer the potential function of ageing-associated miRNAs and to see whether any processes related to ageing or photoageing could be regulated by predicted targets of the validated miRNAs, KEGG pathway enrichment analysis was performed. For this we selected miRNAs whose expression was found to be changed only by sun-exposure (miR-145 miR-6879, miR-3648 and miR-663b) and for those that were found modulated by both sun-exposure and chronological ageing (miR-383, miR-34a and miR-134) (Supplementary Table S2a–g).

For miR-145, signaling pathways playing important roles in epidermal development and skin biology such as the Mitogen-activated protein kinases signaling, TGF-β signaling pathway, tight junctions, vascular endothelial growth factor signaling, Rap1 signaling and regulation of actin cytoskeleton were significantly enriched among its predicted targets in KEGG pathway analysis. Interestingly, miR-145 has been also reported as a regulator of skin pigmentation^23^ (Supplementary Table S2a).

Pathways crucial for cellular differentiation, development, and metabolism, such as adherens junction, PPAR signaling and alpha-Linolenic acid metabolism were enriched among the predicted targets of miR-6879. Among the predicted targets of miR-663b and miR-3648, signaling pathways regulating pluripotency of stem cells, cell adhesion molecule, cytosolic DNA-sensing pathway and RIG-I-like receptor signaling pathway were significantly enriched (Supplementary Table S2b–d).

Predicted miR-383-target genes were significantly enriched in KEGG pathways involved in apoptosis, RNA degradation, AMP-activated protein kinase signaling and p53 signaling pathway, which has previously been shown to be associated with photoageing^24^ suggesting that miR-383 up-regulation could play a part in regulating these ageing-related signaling pathways and that the altered level of the miRNA in sun-exposed skin could contribute to the molecular, cellular and histological alterations associated with photoageing (Supplementary Table S2e).

KEGG-pathway analysis found that adherens junction, MAPK, Notch signaling were the top enriched KEGG terms among the predicted targets of miR-34a, which is in accordance with its well-established role as a tumor suppressor (Supplementary Table S2f).

Skin function and ageing-associated inflammation genes regulating longevity, NF-кB signaling, ErbB signaling, TNF signaling and toll-like receptor signaling were enriched among the predicted targets of miR-134, suggesting that it has important function in skin ageing and inflammation (Supplementary Table S2g).

Because we observed the enrichment of genes involved in p53 and p63 signaling pathways, and because these genes are key regulators of epidermis development, cell proliferation, differentiation and apoptosis, we next retrieved expression data for tumor protein P53 (TP53), tumor protein P63 (TP63), as well as p53-target genes MDM2 and proliferating cell nuclear antigen (PCNA) from the Genotype-Tissue Expression (GTEx) Portal (https://www.gtexportal. org/home/) containing expression data from sun-protected (n = 250) and sun-exposed (n = 356) skin samples. Analysis of gene expression data revealed no significant changes in p53 expression and its target gene MDM2 in the sun-exposed skin (Supplementary Figure S3). Interestingly, we observed a significant downregulation of the master regulator of skin development and homeostasis, TP63 in sun-exposed skin (Supplementary Figure S3). Notably, we also observed downregulation of TP53 target gene PCNA, which is in line with the decreased proliferative capacity of the cells in ageing skin (Supplementary Figure S3).

## Discussion

The present study explored the impact of photoageing and chronological ageing on the miRNA signature of human skin. While photoageing had a robust effect on the skin miRNome, only a mild effect of chronological ageing was observed on the miRNome of human skin. We identified and validated miRNAs, which were altered in sun-exposed skin samples only (photoageing-associated miRNAs: miR-145, miR-6879, miR-3648 and miR-663b) or deregulated both by chronological and photoageing (miR-383 miR-34a and miR-134). KEGG pathway enrichment for the predicted targets genes for these miRNAs revealed the enrichment of signaling pathways important for epidermal function, cellular proliferation, differentiation and senescence, p53 signaling, tight and adherens junction signaling, stem cell regulation, longevity, apoptosis, circadian rhythm, MAPK signaling, NF-кB signaling,TGFβ signaling, and Notch signaling.

p53 has been shown to be an important inducer of ageing in different organisms. Mice with the constitutive activation of p53 have shown an ageing phenotype of epidermal thinning and loss of sebaceous gland activity^25^. Furthermore, the intracellular level of p53 was reported to be reduced during replicative senescence in keratinocytes^26^. Interestingly, two of the miRNAs whose increased expression was validated in photoaged skin, miR-34a and miR-383, are involved in the regulation of the p53-pathway. miR-34 is a part of the p53-gene network and one of the most extensively studied miRNAs. miR-34 acts as a tumor suppressor causing cell cycle G1 phase arrest and inhibits cell cycle regulators cyclin D1 and Cdk4 leading to senescence in primary keratinocytes and embryonic skin^27^. The transcriptional repression of miR-34a along with its family member miR-34c has been also associated with p63-mediated cell cycle progression in epidermal cells^27^. One member of the p53 tumor suppressor gene family is p63 - a key regulator of epidermis development and keratinocyte differentiation^28,29^. We observed the decreased expression of TP63 in sun-exposed skin, which could explain the histopathological events of photoageing, such as thinning of the epidermis alongside with decreased stemness and regenerative capacity of aged skin. Recently, an *in-vivo* study revealed that members of miR-34 family were upregulated in chronologically aged human dermis compared to young^17^. The p53 signaling pathway was also enriched among the predicted targets of miR-383, whose expression was increased in photoaged skin samples. These data suggest that both miR-383 and miR-34a could play an important role in regulating p53-ageing-network, cell cycle, senescence and apoptosis.

The number of β1 integrin positive keratinocytes decreases during skin ageing^30,31^. We observed that the signaling pathway regulating pluripotency of stem cells was enriched among the predicted targets of miR-663b which was found downregulated in photoageing in all 3 age groups. Moreover, miR-663b has been shown to promote cell proliferation, migration and invasion by targeting tumor suppressor candidate 2 in nasopharyngeal carcinoma cell lines^32^.

Tight junctions in the epidermis are essential for barrier function, homeostasis and structure. Chronic UV-exposure reduces tight junctions in the epidermis primarily by affecting the localization of claudins and occludins which results in poor barrier function and water loss^33^. We found that miR-145 was upregulated in the sun-exposed skin of all ages and genes regulating tight junctions were enriched among the predicted targets of this miRNA. Skin barrier function and epidermal homeostasis are key in both extrinsic and intrinsic skin ageing and miR-145 may regulate these functions in ageing skin. Moreover, miR-145 plays an important role in regulating skin pigmentation and positively regulates TGF-β1-induced differentiation of skin myofibroblast in hypertrophic scarring^23,34^.

Chronic inflammation is associated with ageing and characterized by the increased production of pro-inflammatrory mediators such as IL-1β, TNF-α and IL-8 and the activation of NF-кB signaling, as well as infiltration of inflammatory cells^35,36^. Genes regulating NF-кB signaling were eniched among the predicted targets of miR-134, which was found upregulated during chronological and photoageing. Overexpression of miR-134 in hepatic stellate cells increased phosphorylation of NF-кB leading to activation of downstream cascade^37^. In brain, expression of miR-134 has been shown to be limited by SIRT1, a gene regulating longevity, ageing and synaptic plasticity^38^. These findings suggest that miR-134 could play a part in regulating longevity and ageing-associated inflammation.

In contrast to the results presented here, a recent miRNA expression profiling-study comparing sun-exposed and mildly photo-damaged skin of 8 healthy volunteers (average age 65 years), failed to show any significant differences in miRNAs expression^39^. A potential explanation for this discrepancy could be the use of different microarray platform and sample cohort. For our study, donors were selected on strict inclusion and exclusion criteria based on skin folding, photoageing score and phototype thereby limiting the intra-group variability. Results from this study suggest that photoageing has a predominant effect in human skin ageing, while the effects of chronological ageing were less prominent. This may partly be due to the higher power of pair-wise comparisons that were used to detect differentially expressed miRNAs between sun-exposed and sun-protected skin samples and partly it may reflect the strong effect of UV-irradiation-induced genetic and epigenetic changes in the skin^40^.

Taken together, our exploratory study revealed dynamic changes in miRNA expression during chronological and photoageing of the human skin. Many of the identified miRNAs have been overlooked by previous studies on skin ageing-associated miRNAs, which used either cultured skin cells or artificial models of ageing. An important strength of this study is the use of carefully matched/paired biopsies from sun-exposed and sun-protected zones of anatomically close locations from the same individual, limiting the inter-individual variability and allowing the identification of differences in miRNA signature. A limitation of our study is that the functional relevance of the identified changes was not investigated, these results provide a basis for further mechanistic studies into the ageing process of human skin and associated pharmacology.

## Methods

### Volunteers and skin samples

Forty-five Caucasian women were involved in the study after giving their informed consent. The study was approved by the regional ethics committee (Regionala etikprövningsnämnden, Stockholm, Sweden (Regional Ethics Testing Board)) and performed according to the Declaration of Helsinki Principles. Healthy volunteers were distributed in 3 homogenous groups of 15 volunteers each: age group #1 (18–25 y, D01-D15), age group #2 (40–50 y, D31-D45), age group #3 (>70 y, D16-D30). For each volunteer, 3 mm punch biopsies were taken on the height of the internal face of the arm (sun-protected) and in the external bottom of the face of the arm (sun-exposed) under local anesthesia with 1% xylocain. Biopsies were placed in tubes for freezing, using sterile single-use tweezers, then stored at −80 °C until analysis. Table 1 and Supplementary Table S1 summarize the characteristics of volunteers. For detailed inclusion and exclusion criteria, see supplementary methods.

### Total RNA extraction and microarray analysis

Skin biopsies were homogenized using mikro-dismembrator S (Sartorius, Goettingen, Germany) and total RNA was isolated using miRNeasy kit (Qiagen, Stockholm, Sweden). RNA quality and quantity were assessed using Agilent 2100 Bioanalyzer chip (Agilent, Stockholm, Sweden). RNA was hybridized with Affymetrix array chip (Gene Titan microRNA 4.1) containing 2578 human miRNAs probes and miRNome profiling was performed. Microarray data was analyzed using Affymetrix standard protocol and fold change, p-value and q-values were calculated using SAM (significance analysis for microarray), MEV (Multiple Experiment Viewer) (TM4). Microarray data is available on NCBI Gene Expression Omnibus (GSE114006).

### qRT-PCR

Quantification of miRNAs by TaqMan® qRT-PCR was carried out as described by the manufacturer (Thermo Fisher Scientific, Stockholm, Sweden). Briefly, RNA was reverse transcribed using the TaqMan® MicroRNA Reverse Transcription Kit (Thermo Fisher Scientific, Stockholm, Sweden) and miRNA-specific primers (Thermo Fisher Scientific, Stockholm, Sweden). Expression was analyzed using Quantstudio 7 Flex (Thermo Fisher Scientific, Stockholm, Sweden)and expression was normalized against U48 snRNA expression (Thermo Fisher Scientific, Stockholm, Sweden).

### KEGG pathway analysis

 Top 200 mRNA targets were predicted for miR-383, miR-145, miR-6879, miR-34a, miR-663b and miR-3648 using TargetScan 7.1 (Whitehead Institute for Biomedical Research, Cambridge, USA) based on the context score. These predicted mRNA targets were then used to perform KEGG pathway enrichment-analysis using Enrichr (Ma’ayan Laboratory, New York USA). KEGG pathway terms were sorted on p-value (p < 0.05).

### Statistics

Two-class unpaired group comparisons were performed in MEV (TM4) for the microarray data. Mann-Whitney U test was performed using Prism 6.0 (GraphPad, La Jolla, USA) for all the qRT-PCR expression analysis. Calculated p-values were denoted with asterisk symbol on the graphs (*p < 0.05; **p < 0.01; ***p < 0.001; ****p < 0.0001).

## Electronic supplementary material

Supplementary Information
