# Supplementary material for: Identification of chronological and photoageing-associated microRNAs in human skin
Source: Sci Rep. 2018 Aug 28;8:12990. doi: 10.1038/s41598-018-31217-8 (PMC6113407; doi:10.1038/s41598-018-31217-8)
Supplement: Supplementary file 1 — Supplementary Information [file 41598_2018_31217_MOESM1_ESM.pdf]

Identification of chronological and photoageing-associated microRNAs in human skin

Ankit Srivastava[1], Magnus Karlsson[1 3], Claire Marionnet[2], Françoise Bernerd[2], Audrey Gueniche[2], Charles El Rawadi[2], Mona Ståhle[1 3], Enikö Sonkoly[1 3], Lionel Breton[2] and Andor Pivarcsi[1*]

[1]Department of Medicine, Dermatology and Venereology Unit, Karolinska Institutet, Stockholm, Sweden

[2]L'Oréal Research and Innovation, Aulnay-sous-Bois, France

[3]Unit of Dermatology, Karolinska University Hospital, Stockholm, Sweden

***Correspondence**: Andor Pivarcsi, Dermatology and Venereology Unit, Department of Medicine Solna, Karolinska Institutet, CMM L8:02, 17176 Stockholm, Sweden.

Phone: +46 8 51772158. E-mail: andor.pivarcsi@ki.se

Supplementary methods

Inclusion and exclusion criteria

Women in the Young and Middle age groups had a regular cycle of 28 days under efficient contraception (except donor D43). The women over 70 in the Aged group were menopausal for at least 2 years. All volunteers were Phototypes II to III according to Fitzpatrick classification. Colorimetric measures were performed on cheeks with a Chromameter (Minolta CR400) using L*a*b*color system ("Commission Internationale de l'Eclairage", CIE, 1976). The ITA° angle was calculated according to the following formula: ITA°=[ArcTangent((L*-50)/b*)]180/π[1]. The ITA values were comprised between 33-47 corresponding to Very Light-Light-Intermediate skin color groups [2]. BMI values were < 27.0 $Kg.m^{-2}$. For each subject, measures of the skin folding grade were performed on the front upper arm using Densiscore® according to the method described in [3], which consists of compressing the skin horizontally and quantifying the width of the folds generated. The skin folding grade using Densiscore® were ≤ 2 for group #1 , 3-4 for group #2 and ≥ 6 for group #3 which was in agreement with thinner and numerous folds in a young (age group #1) population and wider but fewer folds in an old (age group #3) population. In addition, a photo-ageing score was performed on external arm using a 1-9 photo-ageing photographic scale according to McKenzie et al, score 1 being the lowest photo-ageing grade and score 9 the highest [4]. The mean photo-damage score was 1-3 for the Young group, 4-5 for the Middle age group and 6-8 for the Aged group. Densiscore® grade and photo-aging score allowed us to have volunteers that have biological age close to their age. Immuno-compromised subjects, with a chronic systemic or skin disease as subjects with a history of skin cancer, malignant melanoma were not included in the study. Subjects having exposed themselves to the sun or UV radiation in an excessive way in the previous months were also excluded.

References

1 Chardon, A., Cretois, I. & Hourseau, C. Skin colour typology and suntanning pathways. *Int J Cosm Sc* **13**, 191-208 (1991).

2 Del Bino, S. & Bernerd, F. Variations in skin colour and the biological consequences of ultraviolet radiation exposure. *Br. J. Dermatol* **169 Suppl 3**, 33-40 (2013).

3 Batisse, D., Bazin, R., Baldeweck, T., Querleux, B. & Leveque, J. L. Influence of age on the wrinkling capacities of skin. *Skin Res. Technol* **8**, 148-154 (2002).

4 McKenzie, N. E. *et al.* Development of a photographic scale for consistency and guidance in dermatologic assessment of forearm sun damage. *Arch. Dermatol* **147**, 31-36 (2011).

Supplementary figure and table legends

Supplementary Figure S1. Identification of differentially expressed miRNAs in photoageing. miRNA profiling identified deregulated miRNAs in the sun-exposed skin of young (age group #1, 18-25 years) and aged donors (age group #3, >70 years). (a-b) Heatmap and the table displaying the differentially expressed miRNAs in sun-exposed skin of age groups #1 and #3.

Supplementary Figure S2. Localization of miR-383, miR-145, miR-34a and miR-134 in the skin. FANTOM5 miRNAS atlas was analyzed for the expression of miR-383, miR-145, miR-34a and miR-134 in fibroblast and keratinocytes. Expression (counts per million) was plotted for these miRNAs in fibroblast and keratinocytes.

Supplementary Figure S3. Impact of sun-exposure on TP53, TP63, MDM2 and PCNA. The expression of TP53, TP63, and TP53 target genes MDM2 and PCNA was analyzed in sun-protected (suprapubic) and sun-exposed (lower leg) skin using GTEx portal.

Supplementary Table S1. Detailed donor information. Table describing age, elasticity, pigmentation and photoageing of all the volunteers included in the study.

Supplementary Table S2. Significantly enriched KEGG pathways for the qPCR-validated miRNAs.

KEGG pathway term enrichment analysis was performed using EnrichR, among the predicted targets of all validated miRNAs. (a-g) All significantly enriched pathways are depicted in the tables.

Supplementary Figure S1.

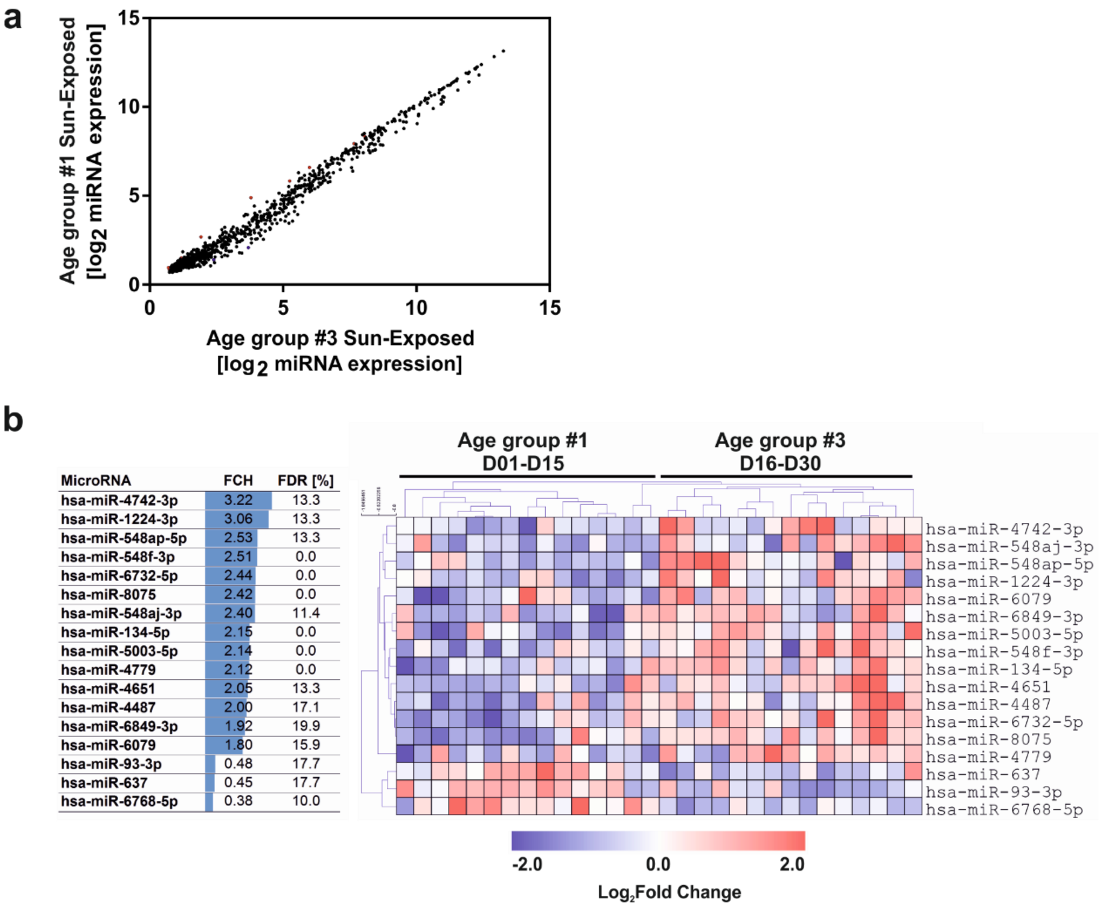

MicroRNA	FCH	FDR [%]
hsa-miR-4742-3p	3.22	13.3
hsa-miR-1224-3p	3.06	13.3
hsa-miR-548ap-5p	2.53	13.3
hsa-miR-548f-3p	2.51	0.0
hsa-miR-6732-5p	2.44	0.0
hsa-miR-8075	2.42	0.0
hsa-miR-548aj-3p	2.40	11.4
hsa-miR-134-5p	2.15	0.0
hsa-miR-5003-5p	2.14	0.0
hsa-miR-4779	2.12	0.0
hsa-miR-4651	2.05	13.3
hsa-miR-4487	2.00	17.1
hsa-miR-6849-3p	1.92	19.9
hsa-miR-6079	1.80	15.9
hsa-miR-93-3p	0.48	17.7
hsa-miR-637	0.45	17.7
hsa-miR-6768-5p	0.38	10.0

Supplementary Figure S2.

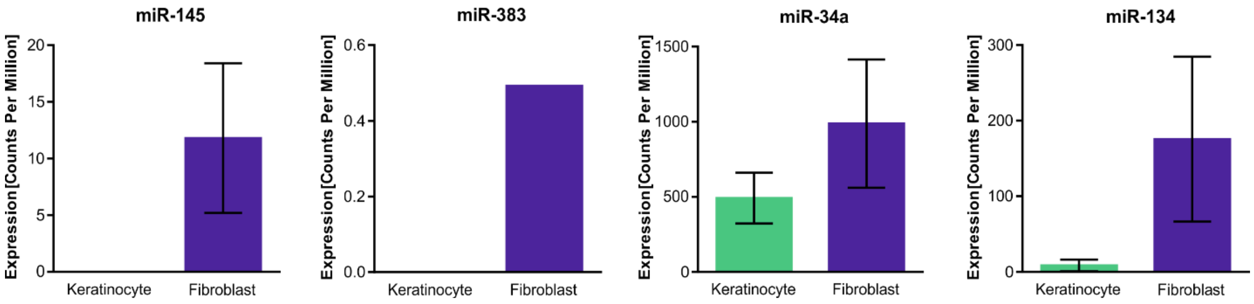

Supplementary Figure S3.

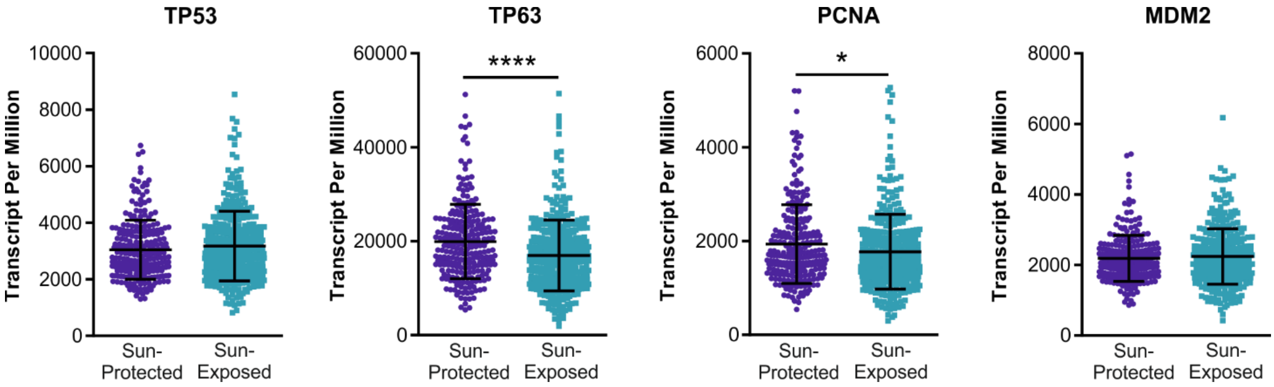

Supplementary Table S1. Donor information

		Age	ITA	Skin Fold Score	Photoaging Score	Menopaus
Age group #1	D1	21	37	1	1	no
	D2	23	41	1	2	no
	D3	22	39	1	1	no
	D4	23	41	1	1	no
	D5	19	40	1	1	no
	D6	24	47	1	2	no
	D7	19	33	1	1	no
	D8	24	37	1	1	no
	D9	24	36	1	1	no
	D10	23	40	1	1	no
	D11	20	39	1	1	no
	D12	21	40	1	1	no
	D13	22	30	1	1	no
	D14	24	36	1	2	no
	D15	25	33	1	1	no
	Average	22.3	37.9	1	1.2	
	SD	1.91	4.1		0.4	

		Age	ITA	Skin Fold Score	Photoaging Score	Menopaus
Age group #2	D31	44	33	3	4	no
	D32	45	34	3	4	no
	D33	42	39	3	4	no
	D34	45	39	3	4	no
	D35	44	45	3	5	no
	D36	43	43	3	4	no
	D37	50	39	3	4	no
	D38	45	36	3	4	no
	D39	44	38	3	4	no
	D40	42	39	3	4	no
	D41	43	38	3	5	no
	D42	40	33	3	4	no
	D43	50	36	3	4	yes
	D44	45	40	3	5	no
	D45	45	36	3	4	no
	Average	44.5	37.9	3	4.2	
	SD	2.67	3.38		0.4	

Age group #3		Age	ITA	Skin Fold Score	Photoaging Score	Menopaus
	**D16**	78	37	>6	6	yes
	**D17**	75	37	>6	6	yes
	**D18**	81	38	>6	6	yes
	**D19**	76	36	>6	6	yes
	**D20**	71	38	>6	6	yes
	**D21**	76	37	>6	6	yes
	**D22**	77	38	>6	6	yes
	**D23**	76	40	>6	7	yes
	**D24**	71	38	>6	6	yes
	**D25**	71	41	>6	6	yes
	**D26**	71	42	>6	7	yes
	**D27**	70	37	>6	6	yes
	**D28**	72	37	>6	6	yes
	**D29**	79	40	>6	7	yes
	**D30**	70	31	>6	6	yes
	**Average**	74.3	37.8	>6	6.2	
	**SD**	3.61	2.54		0.4	

Supplementary Table S2. KEGG pathway enrichment

a. Significantly enriched KEGG pathways among the predicted targets of miR-145

Term	P-value	Z-score
Vibrio cholerae infection_Homo sapiens_hsa05110	0.004	-1.88
Hypertrophic cardiomyopathy (HCM)_Homo sapiens_hsa05410	0.003	-1.82
Bacterial invasion of epithelial cells_Homo sapiens_hsa05100	0.003	-1.79
Dilated cardiomyopathy_Homo sapiens_hsa05414	0.005	-1.78
Axon guidance_Homo sapiens_hsa04360	0.004	-1.70
Proteoglycans in cancer_Homo sapiens_hsa05205	0.010	-1.83
MAPK signaling pathway_Homo sapiens_hsa04010	0.011	-1.79
Oxytocin signaling pathway_Homo sapiens_hsa04921	0.011	-1.74
Hippo signaling pathway_Homo sapiens_hsa04390	0.010	-1.59
Shigellosis_Homo sapiens_hsa05131	0.008	-1.56
TGF-beta signaling pathway_Homo sapiens_hsa04350	0.019	-1.57
Tight junction_Homo sapiens_hsa04530	0.025	-1.44
Endocytosis_Homo sapiens_hsa04144	0.034	-1.62
Focal adhesion_Homo sapiens_hsa04510	0.033	-1.60
Glutathione metabolism_Homo sapiens_hsa00480	0.025	-1.34
Rap1 signaling pathway_Homo sapiens_hsa04015	0.039	-1.56
Regulation of actin cytoskeleton_Homo sapiens_hsa04810	0.041	-1.50
VEGF signaling pathway_Homo sapiens_hsa04370	0.037	-1.45
Serotonergic synapse_Homo sapiens_hsa04726	0.045	-1.39
Epithelial cell signaling in Helicobacter pylori infection_Homo sapiens_hsa05120	0.049	-1.25

b. Significantly enriched KEGG pathways among the predicted targets of miR-6879

Term	P-value	Z-score
Inflammatory bowel disease (IBD)_Homo sapiens_hsa05321	0.019	-1.98
Non-alcoholic fatty liver disease (NAFLD)_Homo sapiens_hsa04932	0.040	-1.81
Adherens junction_Homo sapiens_hsa04520	0.026	-1.77
PPAR signaling pathway_Homo sapiens_hsa03320	0.022	-1.66
alpha-Linolenic acid metabolism_Homo sapiens_hsa00592	0.021	-1.61
SNARE interactions in vesicular transport_Homo sapiens_hsa04130	0.036	-1.55
Asthma_Homo sapiens_hsa05310	0.031	-1.38

c. Significantly enriched KEGG pathways among the predicted targets of miR-663b

Term	P-value	Z-score
Retinol metabolism_Homo sapiens_hsa00830	0.004	-1.85
Viral carcinogenesis_Homo sapiens_hsa05203	0.014	-1.93
Systemic lupus erythematosus_Homo sapiens_hsa05322	0.010	-1.72
HTLV-I infection_Homo sapiens_hsa05166	0.038	-1.84
Alcoholism_Homo sapiens_hsa05034	0.030	-1.83
Signaling pathways regulating pluripotency of stem cells_Homo sapiens_hsa04550	0.049	-1.68
Cell adhesion molecules (CAMs)_Homo sapiens_hsa04514	0.049	-1.61

d. Significantly enriched KEGG pathways among the predicted targets of miR-3648

Term	P-value	Z-score
Viral carcinogenesis_Homo sapiens_hsa05203	0.020	-1.982
Hepatitis C_Homo sapiens_hsa05160	0.029	-1.812
Systemic lupus erythematosus_Homo sapiens_hsa05322	0.030	-1.697
Cytosolic DNA-sensing pathway_Homo sapiens_hsa04623	0.041	-1.627
Shigellosis_Homo sapiens_hsa05131	0.042	-1.599
RIG-I-like receptor signaling pathway_Homo sapiens_hsa04622	0.048	-1.564

e. Significantly enriched KEGG pathways among the predicted targets of miR-383

Term	P-value	Z-score
Prolactin signaling pathway_Homo sapiens_hsa04917	0.006	-1.98
Apoptosis_Homo sapiens_hsa04210	0.013	-1.91
RNA degradation_Homo sapiens_hsa03018	0.008	-1.84
Renal cell carcinoma_Homo sapiens_hsa05211	0.029	-1.78
Protein processing in endoplasmic reticulum_Homo sapiens_hsa04141	0.027	-1.74
AMPK signaling pathway_Homo sapiens_hsa04152	0.036	-1.61
p53 signaling pathway_Homo sapiens_hsa04115	0.033	-1.60
Biosynthesis of amino acids_Homo sapiens_hsa01230	0.039	-1.48
FoxO signaling pathway_Homo sapiens_hsa04068	0.044	-1.57
Circadian rhythm_Homo sapiens_hsa04710	0.038	-1.34
Glyoxylate and dicarboxylate metabolism_Homo sapiens_hsa00630	0.034	-1.18

f. Significantly enriched KEGG pathways among the predicted targets of miR-34a

Term	P-value	Z-score
Adherens junction_Homo sapiens_hsa04520	0.001	-1.84
Other types of O-glycan biosynthesis_Homo sapiens_hsa00514	0.000	-1.59
Galactose metabolism_Homo sapiens_hsa00052	0.004	-1.56
MAPK signaling pathway_Homo sapiens_hsa04010	0.012	-1.90
Bacterial invasion of epithelial cells_Homo sapiens_hsa05100	0.008	-1.70
Notch signaling pathway_Homo sapiens_hsa04330	0.013	-1.54
Retrograde endocannabinoid signaling_Homo sapiens_hsa04723	0.018	-1.77
Arrhythmogenic right ventricular cardiomyopathy (ARVC)_Homo sapiens_hsa05412	0.037	-1.58
Hypertrophic cardiomyopathy (HCM)_Homo sapiens_hsa05410	0.049	-1.56
Cardiac muscle contraction_Homo sapiens_hsa04260	0.043	-1.54

g. Significantly enriched KEGG pathways among the predicted targets of miR-134

Term	P-value	Z-score
Biosynthesis of amino acids_Homo sapiens_hsa01230	0.001	-1.772
Viral carcinogenesis_Homo sapiens_hsa05203	0.002	-1.957
Hepatitis B_Homo sapiens_hsa05161	0.005	-1.885
Alanine, aspartate and glutamate metabolism_Homo sapiens_hsa00250	0.007	-1.617
Ubiquinone and other terpenoid-quinone biosynthesis_Homo sapiens_hsa00130	0.008	-0.677
Regulation of actin cytoskeleton_Homo sapiens_hsa04810	0.008	-1.797
Toxoplasmosis_Homo sapiens_hsa05145	0.009	-1.764
Neurotrophin signaling pathway_Homo sapiens_hsa04722	0.010	-1.699
Cysteine and methionine metabolism_Homo sapiens_hsa00270	0.014	-1.489
ErbB signaling pathway_Homo sapiens_hsa04012	0.015	-1.633
Nitrogen metabolism_Homo sapiens_hsa00910	0.016	-0.439
NF-kappa B signaling pathway_Homo sapiens_hsa04064	0.018	-1.454
Longevity regulating pathway - mammal_Homo sapiens_hsa04211	0.019	-1.574
Epstein-Barr virus infection_Homo sapiens_hsa05169	0.021	-1.627
Arginine biosynthesis_Homo sapiens_hsa00220	0.022	-0.397
Estrogen signaling pathway_Homo sapiens_hsa04915	0.022	-1.502
Non-small cell lung cancer_Homo sapiens_hsa05223	0.023	-1.428
Acute myeloid leukemia_Homo sapiens_hsa05221	0.024	-1.341
Chagas disease (American trypanosomiasis)_Homo sapiens_hsa05142	0.026	-1.344
Toll-like receptor signaling pathway_Homo sapiens_hsa04620	0.028	-1.386
Proximal tubule bicarbonate reclamation_Homo sapiens_hsa04964	0.028	-0.105
Insulin resistance_Homo sapiens_hsa04931	0.030	-1.305
Colorectal cancer_Homo sapiens_hsa05210	0.030	-1.138
TNF signaling pathway_Homo sapiens_hsa04668	0.031	-1.295

Cholinergic synapse_Homo sapiens_hsa04725	0.032	-1.376
Longevity regulating pathway - multiple species_Homo sapiens_hsa04213	0.033	-1.069
cGMP-PKG signaling pathway_Homo sapiens_hsa04022	0.033	-1.267
Pancreatic cancer_Homo sapiens_hsa05212	0.035	-0.976
Renal cell carcinoma_Homo sapiens_hsa05211	0.035	-0.953
Thyroid cancer_Homo sapiens_hsa05216	0.041	-0.068
Alcoholism_Homo sapiens_hsa05034	0.043	-1.151
Prolactin signaling pathway_Homo sapiens_hsa04917	0.043	-0.926
Chronic myeloid leukemia_Homo sapiens_hsa05220	0.045	-0.890
Axon guidance_Homo sapiens_hsa04360	0.048	-0.929
